# The Alterations and Potential Roles of MCMs in Breast Cancer

**DOI:** 10.1155/2021/7928937

**Published:** 2021-08-24

**Authors:** Xinyu Liu, Ying Liu, Qiangshan Wang, Siqi Song, Lingjun Feng, Chunying Shi

**Affiliations:** ^1^School of Basic Medicine, College of Medicine, Qingdao University, Qingdao 266071, China; ^2^Institute for Translational Medicine, The Affiliated Hospital of Qingdao University, College of Medicine, Qingdao University, Qingdao 266021, China; ^3^Jiaozhou Maternal and Child Health Hospital, Qingdao 266300, China; ^4^Thyroid & Breast Surgery, Hospital of Weifang Medical University, Weifang 261031, China

## Abstract

The minichromosome maintenance (MCM) protein family plays a key role in eukaryotic DNA replication and has been confirmed to be associated with the occurrence and progression of many tumors. However, the expression levels, functions, and prognostic values of MCMs in breast cancer (BC) have not been clearly and systematically explained. In this article, we studied the transcriptional levels of MCMs in BC based on the Oncomine database. Kaplan-Meier plotter was used to analyze prognostic value of MCMs in human BC patients. Furthermore, we constructed a MCM coexpression gene network and performed functional annotation analysis through DAVID to reveal the functions of MCMs and coexpressed genes. The data showed that the expression of MCM2–8 and MCM10 but not MCM1 and MCM9 was upregulated in BC. Kaplan-Meier plotter analysis revealed that high transcriptional levels of MCM2, MCM4–7, and MCM10 were significantly related to low relapse-free survival (RFS) in BC patients. In contrast, high levels of MCM1 and MCM9 predicted high RFS for BC patients. This study suggests that MCM2, MCM4–7, and MCM10 possess great potential to be valuable prognostic biomarkers for BC and that MCM1 and MCM9 may serve as potential treatment targets for BC patients.

## 1. Introduction

Surveys show that breast cancer (BC) patients diagnosed worldwide are increasing, and BC is the most common carcinoma type in the female population [1, 2]. BC can be further divided into four subtypes, including luminal A, luminal B, basal-like, and human epidermal growth factor receptor-2 (HER2) overexpression [3]. Classic clinical prognostic markers, such as progesterone receptor (PR), HER2, and estrogen receptor (ER) have played positive roles in endocrine therapy or targeted therapy in BC patients [4]. Because of the heterogeneity of various tumors, the limitations of the current markers are sensitivity and specificity. Therefore, valuable biomarkers are needed as prognostic predictors to effectively upregulate prognosis and precisely individualized therapy effects.

To date, the roles of minichromosome maintenance (MCM) protein family members identified in human cancers have been widely reported. The MCM family plays important roles in the cell cycle and genome replication, including ten members: serum response factor (SRF, also called MCM1) and MCM2–10 [5, 6]. The MCM2–7 complexes are involved in the formation of the prereplication complex and have helicase activity, which makes the DNA detach and leads to the recruitment of DNA polymerase and the activation of DNA replication [7, 8]. MCMs are also involved in the response of DNA damage [9, 10]. In addition, MCM interacts with cellular tumor antigen p53 binding protein 1 (53BP1) and Rad51, and the consumption of MCM leads to a reduction in 53BP1 and Rad51 foci formed after DNA damage [10, 11]. At present, the overexpression of MCM has been detected in various cancer tissues and cancer cell lines, including squamous cell lung carcinoma [12], kidney cancer [13], prostate carcinoma [14], BC [15], digestive system tumors [16–18], brain tumors [19], and lymphomas [20].

The abnormal expression of MCMs and its relationship with clinicopathological characteristics and prognosis have been partially reported in human BC. However, bioinformatics analysis has not been performed to systematically explore the role of MCMs in BC. Based on online databases, we analyzed the expression patterns, clinicopathological characteristics, functions, and different prognostic values of MCMs in patients with BC. In addition, potential regulatory miRNA-regulating MCMs were screened, contributing to regulating the expression of MCMs in BC and identifying targets of precise treatment for BC patients. Our research helps to strengthen and acknowledge of the roles of the MCMs in BC.

## 2. Materials and Methods

### 2.1. Oncomine Analysis

Oncomine [21, 22] (https://www.oncomine.org/resource/main.html) provides gene data that can be used to reveal the expression of target genes in various cancers. The mRNA expression level of MCMs in cancer samples was compared with normal samples. The threshold of *p* value is 0.05, fold change is 2, and gene rank is top 5%.

### 2.2. GEPIA Analysis

GEPIA [23] (http://gepia.cancer-pku.cn/) can be used to analyze the RNA expression of various cancer and normal tissue samples based on TCGA and GTEx. GEPIA was used to perform the correlation analysis of MCMs in BC.

### 2.3. Survival Analysis

Kaplan-Meier plotter [24] (http://www.kmplot.com/) can be used to predict the impact of target genes on the survival rate of patients with different cancer types. We use it to analyze the prognostic value of MCMs and their regulatory miRNAs in BC.

### 2.4. cBioPortal Analysis

The Invasive Breast Carcinoma database (METABRIC, Nature 2012 and Nat Commun 2016, including 2509 samples) was selected to analyze and construct the cancer genome atlas of MCMs based on cBioPortal [25] (https://www.cbioportal.org). Mutations, putative copy-number alterations from DNA copy, and mRNA expression (microarray) z-scores relative to diploid samples of the genomic profiles were chosen to be analyzed.

### 2.5. STRING Analysis

We used STRING [26] database (https://string-db.org) to establish a protein-protein network that showed the coexpression relationships between MCMs and other nodes.

### 2.6. Function Annotation Analysis

DAVID [27] (https://david.ncifcrf.gov/) was used to analyze the MCMs and coexpressed genes to identify GO terms and to visualize genes on Kyoto Encyclopedia of Genes and Genomes (KEGG) pathway maps. *p* < 0.05 was considered to be statistically significant.

### 2.7. ENCORI Analysis

ENCORI [28] (http://starbase.sysu.edu.cn/panCancer.php) was used to predict miRNAs that regulated MCMs and determine the expression levels of miRNA-regulating MCMs. The parameters used in this research were medium stringency (≥3) and 1 cancer type.

### 2.8. Statistical Analysis

The mRNA expression level of MCMs between BC and normal samples was detected to reveal the statistical difference by Student's *t*-tests. Survival curves of various subtypes in BC patients with different expression level of MCMs were drafted based on log-rank test and hazard ratio (HR) by Kaplan-Meier plotter. *p* < 0.05 was considered to be statistically significant.

## 3. Results

### 3.1. The Transcriptional Levels of MCMs and Clinicopathological Characteristics in BC Patients

**Figure 1:**
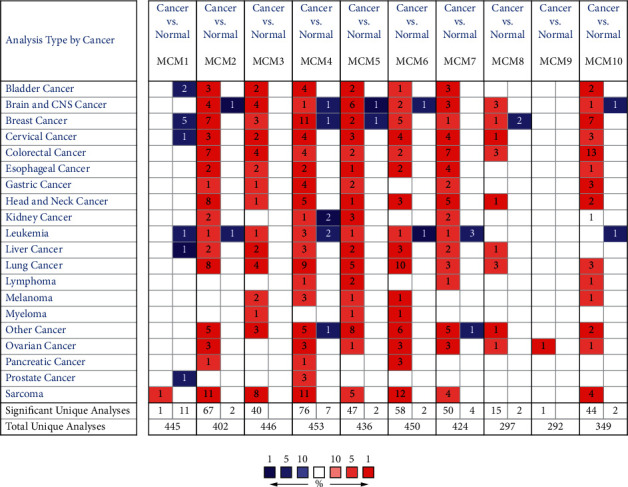
The transcriptional levels of MCMs in various cancers. The figure shows the number of datasets with higher expression levels of MCMs in various types of carcinoma samples compared to normal samples. Red cells represent high mRNA transcriptional level and blue cells represent low mRNA transcriptional level. The threshold of *p* value is 0.05, fold change is 2, and gene rank is top 5%.

**Figure 2:**
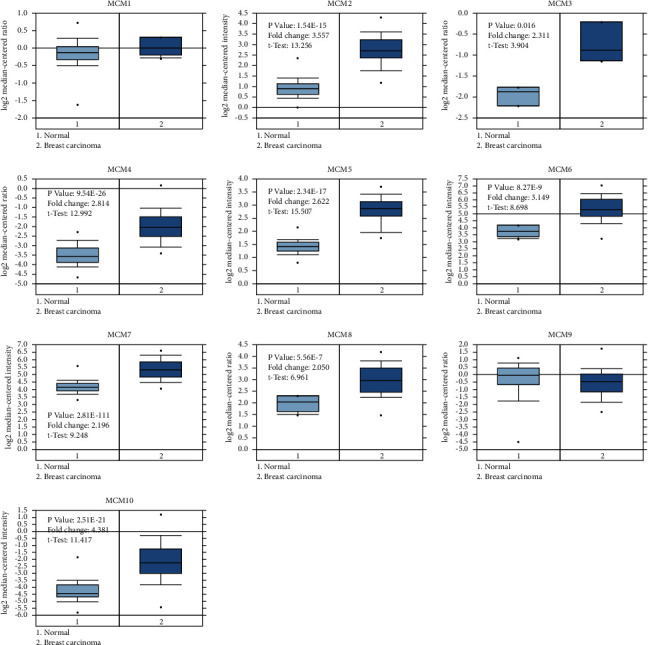
The transcriptional levels of MCMs in BC compared to normal samples based on the Oncomine database.

**Figure 3:**
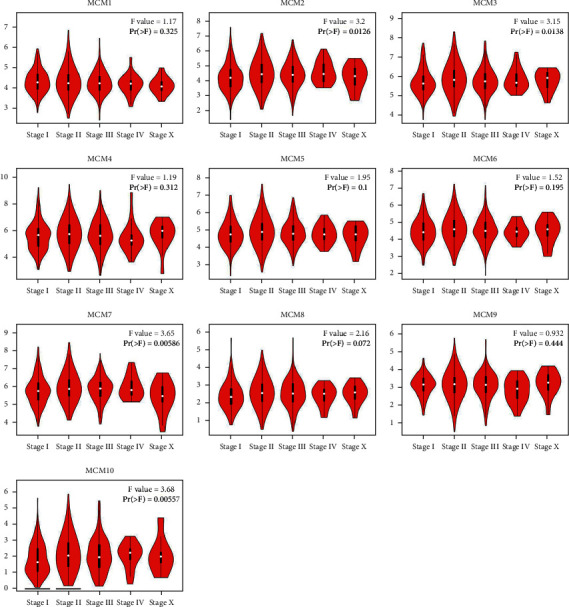
Correlation between MCM expression and tumor stage in breast cancer patients.

Alterations in the transcriptional levels of MCM family members, including MCM1–10, have been widely reported in cancers. Oncomine data showed that the mRNA expression of MCM2–8 and MCM10 but not MCM1 and MCM9 in BC samples was significantly upregulated compared with normal samples (Figures 1 and 2). As shown in Table 1, previous reports indicated that MCM2–8 and MCM10 were upregulated in BC [3, 29–35]. The expression levels of MCMs with tumor stage for breast cancer were analyzed by Gene Expression Profiling Interactive Analysis (GEPIA) database. As shown in Figure 3, MCM2, MCM3, MCM7, and MCM10 groups significantly varied, whereas MCM1, MCM4, MCM5, MCM6, MCM8, and MCM9 groups did not significantly differ.

### 3.2. Diagnostic and Prognostic Value of MCMs in Clinical BC Patients

**Figure 4:**
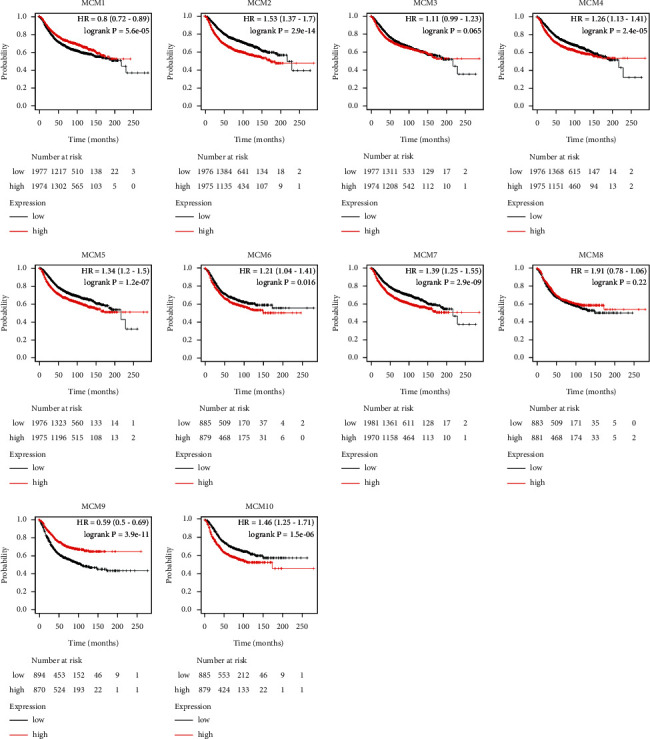
Prognostic value of the expression levels of MCMs in patients with BC (Kaplan-Meier plotter).

Using Kaplan-Meier plotter, we analyzed the prognostic value of MCMs in BC patients and drew related survival maps (Figure 4). Elevated mRNA expression levels of MCM2, MCM4–7, and MCM10 were significantly related to short relapse-free survival (RFS), whereas MCM3 and MCM8 did not, indicating that they were related to poor prognosis in BC patients. However, the decreased expression of MCM1 and MCM9 in BC was significantly related to prolonged RFS, indicating that they were related to good prognosis in BC patients.

### 3.3. Alterations, Gene Correlations, and Coexpression Gene Network of MCMs in BC

**Figure 5:**
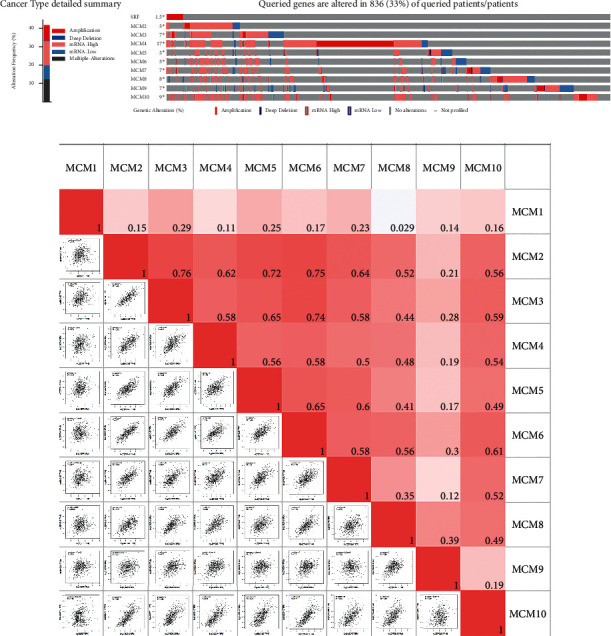
Analysis of alterations and correlation between members of MCMs in BC. (a) Gene expression and alteration analysis of MCMs in BC (cBioPortal). (b) Correlation analysis between different MCMs in BC (GEPIA).

Using the cBioPortal database, we analyzed alterations of MCMs in BC. As shown in Figure 5(a), MCMs were altered in 836 of the 2509 BC samples (33%). The GEPIA database was used to analyze the correlations of the mRNA expressions of MCMs in BC. As shown in Figure 5(b), there were significant positive correlations (*R* > 0.3) between following MCMs: MCM2 with MCM3–8 and MCM10; MCM3 with MCM4–8 and MCM10; MCM4 with MCM5–8 and MCM10; MCM5 with MCM6–8 and MCM10; MCM6 with MCM7, MCM8, and MCM10; MCM7 with MCM8 and MCM10; and MCM8 with MCM9 and MCM10.

**Figure 6:**
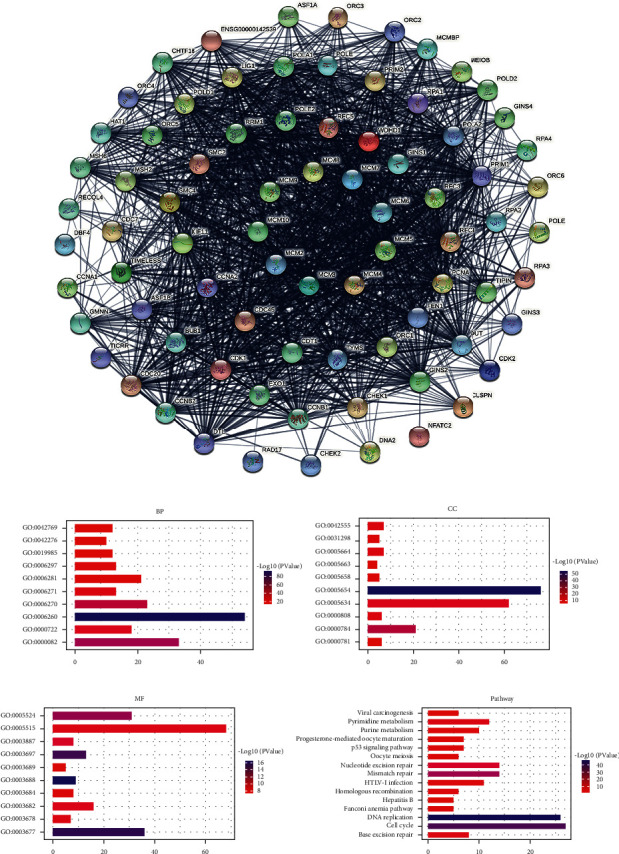
Protein-protein interaction network and functional annotation analysis of MCMs in BC. (a) The protein-protein interaction network of MCMs and frequently altered coexpressed genes (STRING). The potential functions of MCMs and coexpressed genes significantly related to MCMs were predicted based on DAVID and included (b) BP, (c) CC, (d) MF, and (e) KEGG pathways.

Furthermore, we constructed a coexpressed gene network of MCMs (Figure 6(a)). In addition, the potential functions of MCMs and coexpressed genes significantly related to MCMs were predicted by performing Gene Ontology (GO) analysis, including biological processes (BPs), cellular components (CCs), and molecular functions (MFs), and KEGG analysis based on DAVID. We found that GO:0006260 (DNA replication), GO:0000082 (G1/S transition of mitotic cell cycle), and GO:0006270 (DNA replication initiation) were significantly regulated by alterations in MCMs (Figure 6(b)). Moreover, these MCM alterations significantly affected GO:0005654 (nucleoplasm), GO:0000784 (nuclear chromosome, telomeric region), GO:0005634 (nucleus), GO:0005664 (nuclear origin of replication recognition complex), GO:0042555 (MCM complex), GO:0000808 (origin recognition complex), and GO:0005658 (alpha DNA polymerase: primase complex) (Figure 6(c)). Alterations in MCMs also significantly controlled GO:0003688 (DNA replication origin binding), GO:0003677 (DNA binding), GO:0003697 (single-stranded DNA binding), GO:0005524 (ATP binding), GO:0003887 (DNA-directed DNA polymerase activity), GO:0003682 (chromatin binding), GO:0005515 (protein binding), and GO:0003678 (DNA helicase activity) (Figure 6(d)). The important roles of MCMs in DNA replication and cell cycle have been widely recognized.

**Figure 7:**
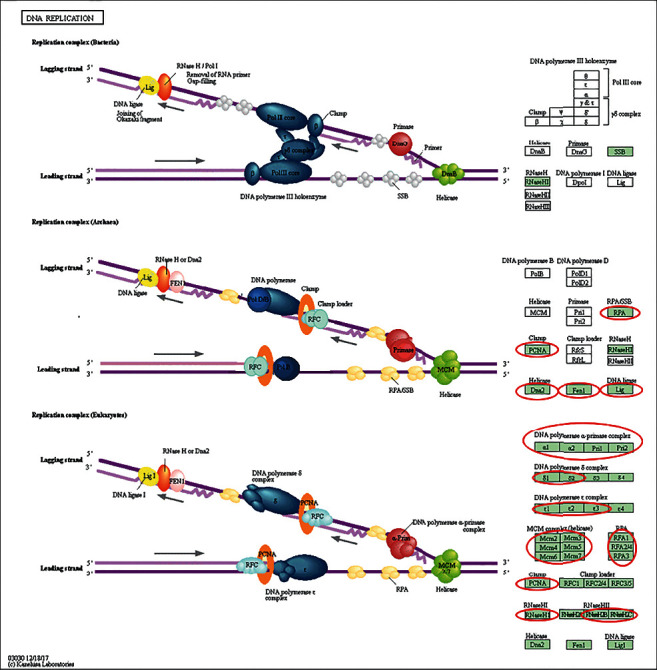
Visualization genes on DNA replication map.

**Figure 8:**
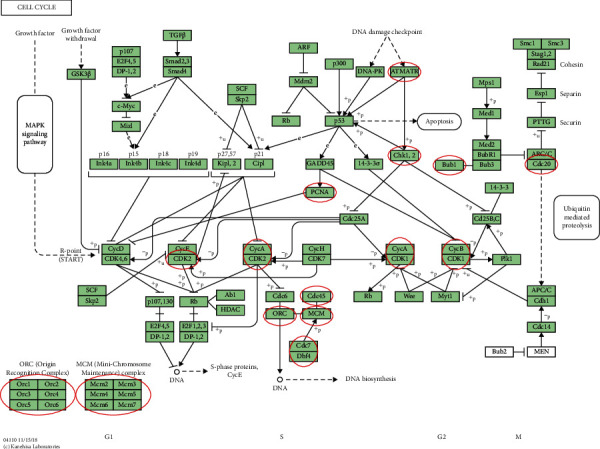
Visualization genes on cell cycle map.

**Figure 9:**
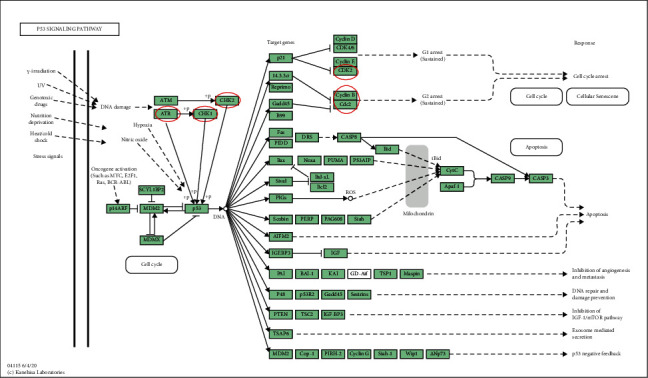
Visualization genes on p53 pathway map.

KEGG analysis was used to define pathways related to the altered functions of MCMs and frequently altered coexpressed genes. Through KEGG analysis, pathways related to the altered functions of MCMs in BC were discovered (Figure 6(e)). Among these pathways, DNA replication, cell cycle, and P53 signaling pathways were significantly related to the occurrence and progression of BC (Figures 7–9).

### 3.4. Regulatory miRNAs and Survival Analysis

**Figure 10:**
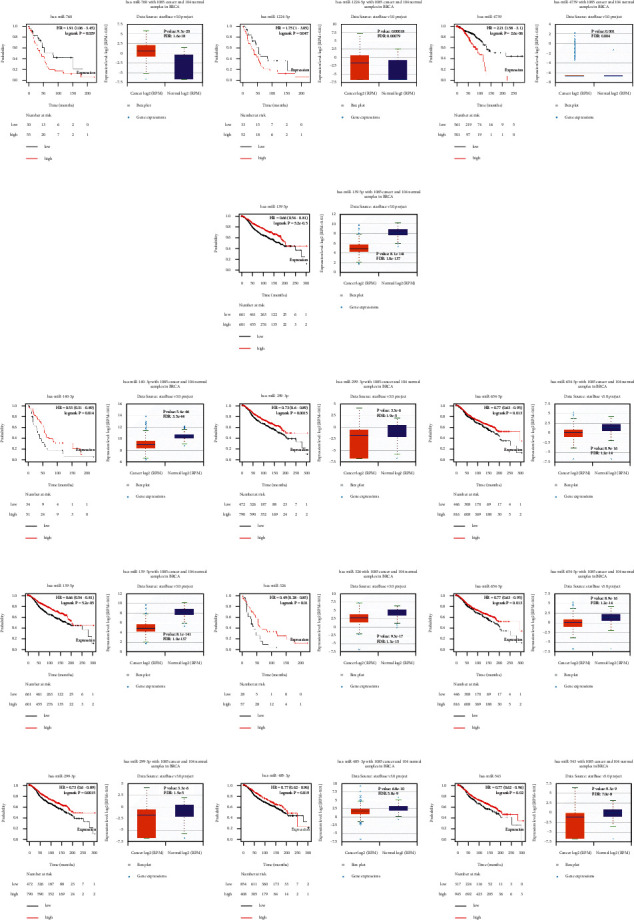
The miRNAs that associated with prognosis in BC patients. (a) has-miR-760, has-miR-1224-5p, and has-miR-4739 predicted poor overall survival in BC patients. (b) has-miR-139-5p predicted good overall survival in BC patients. (c) has-miR-140-3p, has-miR-299-3p, and has-miR-654-5p predicted good overall survival in BC patients. (d) has-miR-139-5p, has-miR-326, and has-miR-654-5p predicted good overall survival in BC patients. (e) has-miR-229-3p, has-miR-485-3p, and has-miR-543 predicted good overall survival in BC patients.

**Figure 11:**
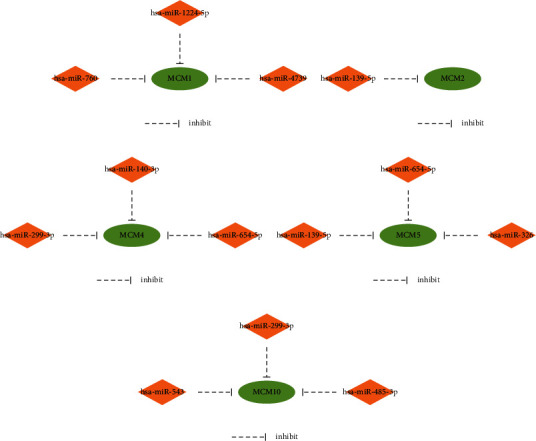
miRNA-mRNA regulation network. (a) miRNA-MCM1 regulation network. (b) miRNA-MCM2 regulation network. (c) miRNA-MCM4 regulation network. (d) miRNA-MCM5 regulation network. (e) miRNA-MCM10 regulation network.

As shown in Tables S1–S8, 137 miRNAs regulating MCM1, 62 miRNAs regulating MCM2, 62 miRNAs regulating MCM4, 41 miRNAs regulating MCM5, 47 miRNAs regulating MCM6, 40 miRNAs regulating MCM7, 9 miRNAs regulating MCM9, and 63 miRNAs regulating MCM10 were predicted with the ENCORI platform. Among them, 45 miRNA-MCM1 pairs, 22 miRNA-MCM2 pairs, 15 miRNA-MCM4 pairs, 15 miRNA-MCM5 pairs, 20 miRNA-MCM6 pairs, 9 miRNA-MCM7 pairs, 6 miRNA-MCM9 pairs, and 23 miRNA-MCM10 pairs were negatively correlated. Furthermore, the ability to predict poor prognosis in BC patients with MCM1 expression was significant for three miRNAs: hsa-miR-760 (*p* value = 0.000825), hsa-miR-1224-5p (*p* value = 0.0135), and hsa-miR-4739 (*p* value = 0.0368) (Figure 10(a)). 10 miRNAs were low expression in BC tissues, and their low expression predicted good prognosis in BC patients. These miRNAs negatively regulated MCM2, MCM4, MCM5, and MCM10 (Figures 10(b)–10(e)). Negative miRNA-MCM pairs were as follows: hsa-miR-139-5p-MCM2 (*p* value = 2.73*E* − 12), hsa-miR-299-3p-MCM4 (*p* value = 4.64*E* − 06), hsa-miR-654-5p-MCM4 (*p* value = 1.28*E* − 07), hsa-miR-140-3p-MCM4 (*p* value = 1.48*E* − 02), hsa-miR-139-5p-MCM5 (*p* value = 7.77*E* − 03), hsa-miR-326-MCM5 (*p* value = 9.16*E* − 03), hsa-miR-654-5p-MCM5 (*p* value = 1.18*E* − 06), hsa-miR-299-3p-MCM10 (*p* value = 7.45*E* − 04), hsa-miR-485-3p-MCM10 (*p* value = 4.55*E* − 02), and hsa-miR-543-MCM10 (*p* value = 1.29*E* − 02). The above results suggest that the established miRNA-MCM regulatory networks may be valuable prognostic markers and therapeutic targets for BC (Figures 11(a)–11(e)).

## 4. Discussion

An imbalance in MCM mRNA expression levels has been reported in many types of cancer [12–20]. Although the roles of MCMs in the occurrence, distant metastasis, and prognosis of patients with various tumors have been partially confirmed, the comprehensive biological information of MCMs in BC has not been systematically clarified. This study analyzed the expression levels, clinicopathological characteristics, functions, and prognostic values of MCMs in BC to aid in the treatment design of BC patients and prognostic accuracy.

The MCM2–8 and MCM10 expression levels were upregulated in BC, but high expression of MCM1 and MCM9 in BC had not been shown. MCM1 (also called SRF) plays an important role in the pathogenesis of human diseases and contributes to the metastasis and colonization of BC cells. It has been reported that the suppressor of cancer cell invasion (SCAI) protein can form a complex with MRTF and SRF to inhibit the invasion of human BC cells [36]. In addition, knocking out MRTFA subtypes or MCM1 reduces the targeted migration and invasion of human BC (MDA-MB-231) cells [37], indicating that MCM1 plays key roles in the distant metastasis of BC. In our research, we found that low expression of MCM1 in BC was closely associated with good prognosis for BC patients.

High MCM2 expression is related to BC with a high histological grade, while low expression level of MCM2 increases the possibility of RFS in patients with BC [38]. Some reports suggest that MCM2 and MCM3 may be used as substitutes for Ki-67 to measure the proliferation of BC cells and predict prognosis [39, 40]. Our study showed that MCM2 was upregulated in BC and its upregulation was significantly associated with poor prognosis for BC patients.

Some studies reported that MCM3 was significantly upregulated in BC and recommended it as a substitute for Ki-67 to measure the proliferation of BC cells and predict prognosis [41, 42]. In our study, we also proved that the MCM3 was overexpressed in BC. However, we did not find a significant relationship between high MCM3 expression and the prognosis of BC patients, which still needs further validation with clinical data.

It has been reported that the MCM4 expression level in clinical samples of BC is significantly higher than that in non-tumor breast epithelium [38, 42]. Shima et al. isolated a subtype mutation of MCM4 called Chaos3 (3 chromosomal aberrations occurring spontaneously). Chaos3 mutations destabilize the MCM2–7 complexes, leading to impaired DNA replication, which increases the risk of BC in the population [43]. We found that MCM4 was overexpressed in BC. In addition, high MCM4 expression was significantly related to shorter RFS in BC patients, suggesting the prognostic value of MCM4 in BC patients. The study of Issac et al. [38] also supports our view.

While the expression of MCM5 has been accurately reported in other cancers, it has not been done so in BC. For example, the high MCM5 expression is associated with the malignant state and a poor prognosis in cervical adenocarcinoma patients and regulates the proliferation of cervical adenocarcinoma cells [44]. Elevated levels of MCM5 in urine sediment can be used to strongly predict bladder cancer [45]. Our research revealed that the MCM5 expression level in BC was upregulated. In addition, high MCM5 expression was significantly related to low RFS in BC patients, indicating the prognostic value of MCM5 in BC patients.

A high mRNA expression level of MCM6 is related to BC with a high histological grade [38]. In our research, we found that MCM6 was significantly increased in BC. In addition, high mRNA expression of MCM6 was significantly related to shorter RFS for BC patients, indicating the prognostic value of MCM6 expression in BC patients. The study of Issac et al. [38] also supports our view.

Huang et al. showed that epidermal growth factor receptor enhanced MCM7-mediated DNA replication through the tyrosine phosphorylation of Lyn kinase in human cancers [46]. Li et al. reported that trifluoropyridine significantly downregulates the expression of PCNA, MCM7, and antiapoptotic Bcl2 in TNBC cells and selectively inhibits the growth of TNBC [47]. Our research found that the expression of MCM7 in BC was upregulated and significantly related to shorter RFS in BC patients, revealing the prognostic value of MCM7 in BC patients.

MCM8 and MCM9 are paralogs of the MCM2–7 replication helicases [48] and are related to HR in mitotic and meiotic cells [49–51]. In addition, MCM8 and MCM9 can cause hematopoietic DNA damage, leading to p53-dependent medullary tumors [52]. However, there is no relevant report in BC. Our research showed that the MCM8 expression level in BC was significantly upregulated. However, no significant correlation between high MCM8 expression and RFS in BC patients was found. In contrast, MCM9 was not upregulated in BC and we found that high MCM9 expression was significantly related to longer RFS in BC patients, indicating that MCM9 has good prognostic value in BC patients.

Currently, it is known that the cell cycle regulation interaction between MCM10 and the dihexamers MCM2–7 is necessary for helicase division and S phase activation [53]. Yang et al. proved that MCM10 promoted the invasion/migration potential of BC cells through Wnt/*β*-catenin signaling and was positively associated with the poor prognosis of BC [54]. In our study, we found that MCM10 was overexpressed in BC and significantly associated with shorter RFS in BC patients, indicating that MCM10 has good prognostic value in BC patients.

Studies have shown that abnormally expressed microRNAs can become a sign of cancer. MicroRNA expression is significantly related to tumor occurrence, progression, and treatment, indicating that they may become prognostic and predictive markers [55]. We identified 13 negatively correlated miRNA-MCM pairs, including hsa-miR-760-MCM1, hsa-miR-1224-5p-MCM1, hsa-miR-4739-MCM1, hsa-miR-139-5p-MCM2, hsa-miR-299-3p-MCM4, hsa-miR-654-5p-MCM4, hsa-miR-140-3p-MCM4, hsa-miR-139-5p-MCM5, hsa-miR-326-MCM5, hsa-miR-654-5p-MCM5, hsa-miR-299-3p-MCM10, hsa-miR-485-3p-MCM10, and hsa-miR-543-MCM10, that may regulate MCM expression in BC and be used to predict the prognosis of BC patients.

Functionally, miRNA-760 inhibits the proliferation and metastasis of BC cells by downregulating NANOG and mediates chemoresistance by inhibiting the epithelial-mesenchymal transition of BC cells. Lv et al. reported that MCF-7 human BC cells overexpressing miR-760 are resistant to Adriamycin [56–58]. In addition, hsa-miR-1224-5p can be used as a valuable treatment target for glioblastoma multiforme [59]. However, the function of hsa-miR-4739 in BC has not been reported. The loss of Opa-interacting protein 5 can inhibit BC proliferation by the miR-139-5p/NOTCH1 pathway [60].

Furthermore, hsa-miR-299-3p may play a key role in thyroid cancer [61]. Hsa-miR-654-5p regulates the osteogenic differentiation of human bone marrow mesenchymal stem cells by inhibiting bone morphogenetic protein 2 [62]. The increased expression of hsa-miR-140-3p 5'isomiR contributes to tumor suppressor effect of hsa-miR-140-3p by reducing the proliferation and migration of BC [63]. Hsa-miR-326 participates in the chemotherapy resistance of BC by regulating the expression of multidrug resistance-related protein 1 [64]. Hsa-miR-326 can inhibit BC by targeting SOX12, making miR-326 a promising therapeutic target for BC [65]. Hsa-miR-543 functions as a carcinoma suppressor in glioma [66]. Moreover, Yu et al. revealed that hsa-miR-543 functions as a tumor suppressor in ovarian carcinoma by targeting TWIST1 [67]. In addition, hsa-miR-543 can target TRPM7 to inhibit cervical cancer [68].

Our findings suggest that MCM family members play crucial roles in BC progression, and MCM2, MCM4–7, and MCM10 in tumor tissues possess great potential to be valuable prognostic biomarkers for BC patients. Clinically, the detection of these protein biomarkers mainly depends on tissue biopsy, which is an invasive method and not always feasible or repeatable. With the development of liquid biopsy, these limitations are being gradually overcome. Liquid biopsy is a rapid, comprehensive, and non-invasive detection method and allows for the longitudinal assessment of cancer evolution. A blood-based liquid biopsy has been reported to efficiently capture circulating tumor cells (CTCs) and circulating tumor-derived nucleic acids, including circulating tumor DNA (ctDNA) [69–72]. CTCs are cancer cells originating from the primary tumor and metastatic sites, which are found in the blood. Increasing evidence shows that CTCs act as valuable biomarkers with high sensitivity and specificity to monitor therapeutic efficacy and predict prognosis in metastatic BC [73, 74]. Due to the crucial role of MCM family members in DNA homeostasis, MCMs are probably involved in the regulation of CTC biological behavior. Therefore, investigating the expression pattern and function of MCMs in different phenotype of CTCs may provide new insight into evaluating the therapeutic efficacy and prognosis of BC patients. ctDNA is released from cancer cells and contains tumor-specific genetic and epigenetic alterations. It has been reported that the mutations or alterations of genes in ctDNA are closely associated with curative effect of therapies in BC [74, 75]. ctDNA analysis may provide an excellent tool to monitor therapeutic efficacy and predict prognosis. For instance, the analysis of ESR1 mutations in ctDNA of metastatic BC patients can be used to predict resistance to endocrine therapy [76]. The detection of PIK3CA alterations in plasma-derived ctDNA and PIK3CA ctDNA levels predicts the response of BC patients to palbociclib and fulvestrant therapy [77]. Moreover, analysis of HER2 mutation frequency in ctDNA can be used to predict response of BC patients to neratinib with high sensitivity and specificity [78]. In addition, ctDNA fraction and somatic copy-number alterations are correlated with significantly worse outcomes in triple-negative BC patients [69, 79]. These studies provide great help for clinicians to adjust the appropriate therapeutic strategy for BC patients in time. Our data from functional annotation analysis revealed that MCMs are involved in the regulation of several pathways, including nucleotide excision repair, mismatch repair, DNA replication, and base excision repair. This means that dysregulation of MCMs might be involved in the occurrence of mutation or alteration of genes in DNA. Future studies are required to investigate the detailed mechanisms of MCMs involved in the occurrence of gene mutation or alteration, which may facilitate the clinical application of ctDNA and development of new therapeutic strategies for BC patients.

## 5. Conclusion

In this study, we systematically analyzed the expression levels, clinicopathological characteristics, functions, and prognostic values of MCMs in BC. Our results suggest that upregulated expression levels of MCM2–8 and MCM10 in BC samples play important roles in BC. High expression of MCM2, MCM4–7, and MCM10 shows great potential to be molecular markers to identify patients with BC. Additionally, MCM1 and MCM9 also exhibit the possibility as prognostic markers for improving the survival rate of BC patients and prognostic accuracy. The miRNAs regulating MCM1, MCM2, MCM4–7, and MCM10 can be involved in carcinogenesis and improve the prognosis of BC patients. However, there are still some limitations in our study. For instance, only Kaplan-Meier plotter was used to evaluate the prognostic value of MCMs in BC. A multivariable Cox model is required to further validate these findings in future study.

## Figures and Tables

**Table 1:** The significant changes of MCM expression in transcription level between different types of breast cancer and normal breast tissues (Oncomine database).

	Type of breast cancer versus normal breast tissue reference invasive breast carcinoma	Fold change	*p* value	*T*-test	Source and/or reference
MCM1	NA	NA	NA	NA	NA
	Medullary breast carcinoma	3.557	1.54*E* − 15	13.256	Curtis breast [29]
	Invasive ductal breast carcinoma	2.503	3.82*E* − 92	34.511	Curtis breast [29]
	Breast carcinoma	2.062	4.52*E* − 6	6.693	Curtis breast [29]
MCM2	Mixed lobular and ductal breast carcinoma	2.200	1.18*E* − 6	8.165	TCGA
	Invasive lobular breast carcinoma	2.120	1.06*E* − 12	8.432	TCGA
	Invasive ductal breast carcinoma	2.877	5.24*E* − 31	17.777	TCGA
	Invasive breast carcinoma	2.184	1.30*E* − 17	9.743	TCGA
	Fibroadenoma	2.311	0.016	3.904	Sorlie breast [3]
MCM3	Fibroadenoma	2.535	0.019	4.415	Sorlie breast 2 [30]
	Medullary breast carcinoma	2.101	1.62*E* − 11	9.579	Curtis breast [21]
	Ductal breast carcinoma	3.081	3.36*E* − 14	13.705	Perou breast [31]
	Lobular breast carcinoma	2.698	0.013	4.040	Perou breast [31]
	Invasive ductal breast carcinoma	3.192	3.89*E* − 48	22.602	TCGA
	Invasive breast carcinoma	2.814	9.54*E* − 26	12.992	TCGA
	Invasive ductal and lobular carcinoma	2.990	2.27*E* − 7	13.999	TCGA
MCM4	Invasive lobular breast carcinoma	2.048	1.02*E* − 13	9.018	TCGA
	Medullary breast carcinoma	3.126	1.96*E* − 13	11.226	Curtis breast [29]
	Invasive ductal breast carcinoma	2.412	1.58*E* − 85	32.416	Curtis breast [29]
	Invasive breast carcinoma	2.011	4.18*E* − 6	5.726	Curtis breast [29]
	Invasive breast carcinoma	2.199	5.37*E* − 6	9.490	Gluck breast [32]
	Ductal breast carcinoma	3.806	7.88*E* − 8	8.590	Richardson breast 2 [33]
MCM5	Medullary breast carcinoma	2.622	2.34*E* − 17	15.507	Curtis breast [29]
	Intraductal cribriform breast adenocarcinoma	2.082	2.53*E* − 4	5.919	TCGA
	Ductal breast carcinoma	3.149	8.27*E* − 9	8.698	Richardson breast 2 [33]
	Medullary breast carcinoma	2.551	1.26*E* − 12	10.679	Curtis breast [21]
MCM6	Invasive lobular breast carcinoma	3.692	0.009	2.828	Radvanyi breast [34]
	Invasive ductal breast carcinoma	3.329	0.012	2.737	Radvanyi breast [34]
	Invasive ductal breast carcinoma	2.358	0.007	3.143	Turashvili breast [35]
MCM7	Medullary breast carcinoma	2.196	2.81*E* − 12	9.248	Curtis breast [29]
MCM8	Ductal breast carcinoma	2.050	5.56*E* − 9	6.961	Richardson breast 2 [33]
MCM9	NA	NA	NA	NA	NA
	Male breast carcinoma	4.716	5.49*E* − 26	20.205	TCGA
	Invasive ductal breast carcinoma	4.774	1.21*E* − 45	21.470	TCGA
	Invasive breast carcinoma	4.381	2.51*E* − 21	11.417	TCGA
MCM10	Invasive lobular breast carcinoma	2.510	4.92*E* − 11	7.582	TCGA
	Ductal breast carcinoma	7.508	1.68*E* − 12	9.492	Richardson breast 2 [33]
	Invasive ductal breast carcinoma	2.077	2.99*E* − 92	29.777	Curtis breast [29]
	Medullary breast carcinoma	3.802	3.90*E* − 13	11.272	Curtis breast [29]

NA, not available; TCGA, the Cancer Genome Atlas.

## Data Availability

The data used to support the findings of this study can be obtained through the corresponding author upon reasonable request.

## Supplementary Materials

Supplementary Materials Table S1: correlation of miRNA-MCM1 pairs identified by ENCORI database. Table S2: correlation of miRNA-MCM2 pairs identified by ENCORI database. Table S3: correlation of miRNA-MCM4 pairs identified by ENCORI database. Table S4: correlation of miRNA-MCM5 pairs identified by ENCORI database. Table S5: correlation of miRNA-MCM6 pairs identified by ENCORI database. Table S6: correlation of miRNA-MCM7 pairs identified by ENCORI database. Table S7: correlation of miRNA-MCM9 pairs identified by ENCORI database. Table S8: correlation of miRNA-MCM10 pairs identified by ENCORI database.
