# Supplementary material for: The Alterations and Potential Roles of MCMs in Breast Cancer
Source: J Oncol. 2021 Aug 24;2021:7928937. doi: 10.1155/2021/7928937 (PMC8407980; doi:10.1155/2021/7928937)
Supplement: Supplementary Materials — Table S1: correlation of miRNA-MCM1 pairs identified by ENCORI database. Table S2: correlation of miRNA-MCM2 pairs identified by ENCORI database. Table S3: correlation of miRNA-MCM4 pairs identified by ENCORI database. Table S4: correlation of miRNA-MCM5 pairs identified by ENCORI database. Table S5: correlation of miRNA-MCM6 pairs identified by ENCORI database. Table S6: correlation of miRNA-MCM7 pairs identified by ENCORI database. Table S7: correlation of miRNA-MCM9 pairs identified by ENCORI database. Table S8: correlation of miRNA-MCM10 pairs identified by ENCORI database. [file 7928937.f1.docx]

**Table S1. Correlation of miRNA-MCM1 pairs identified by ENCORI database.**

| **No.** | **miRNA** | **Coefficient-R** | **P value** |
| --- | --- | --- | --- |
| 1 | hsa-miR-15a-5p | 0.061 | 0.0432 |
| 2 | hsa-miR-22-3p | 0.014 | 0.639 |
| 3 | hsa-miR-23a-3p | 0.072 | 0.0184 |
| 4 | hsa-miR-24-3p | 0.156 | 0.000000229 |
| 5 | hsa-miR-28-5p | 0.258 | 5.46E-18 |
| 6 | hsa-miR-31-5p | 0.137 | 0.00000596 |
| 7 | hsa-miR-95-3p | 0.024 | 0.436 |
| 8 | hsa-miR-101-3p | 0.116 | 0.000123 |
| 9 | hsa-miR-199a-5p | 0.072 | 0.0172 |
| 10 | hsa-miR-199a-3p | 0.016 | 0.599 |
| 11 | hsa-miR-129-5p | 0.171 | 1.49E-08 |
| 12 | hsa-miR-148a-3p | 0.03 | 0.318 |
| 13 | hsa-miR-147a | 0.014 | 0.656 |
| 14 | hsa-miR-181a-5p | 0.142 | 0.00000247 |
| 15 | hsa-miR-181b-5p | 0.169 | 1.96E-08 |
| 16 | hsa-miR-181c-5p | 0.152 | 0.00000046 |
| 17 | hsa-miR-199b-5p | 0.103 | 0.000673 |
| 18 | hsa-miR-214-3p | 0.095 | 0.00165 |
| 19 | hsa-miR-224-5p | 0.208 | 4.54E-12 |
| 20 | hsa-miR-15b-5p | 0.033 | 0.279 |
| 21 | hsa-miR-122-5p | 0.011 | 0.72 |
| 22 | hsa-miR-125b-5p | 0.222 | 1.52E-13 |
| 23 | hsa-miR-130a-3p | 0.262 | 1.7E-18 |
| 24 | hsa-miR-138-5p | 0.284 | 1.65E-21 |
| 25 | hsa-miR-142-5p | 0.111 | 0.000265 |
| 26 | hsa-miR-144-3p | 0.087 | 0.004 |
| 27 | hsa-miR-152-3p | 0.014 | 0.65 |
| 28 | hsa-miR-9-5p | 0.19 | 2.61E-10 |
| 29 | hsa-miR-146a-5p | 0.198 | 4.27E-11 |
| 30 | hsa-miR-150-5p | 0.192 | 1.91E-10 |
| 31 | hsa-miR-154-5p | 0.053 | 0.0815 |
| 32 | hsa-miR-185-5p | 0.037 | 0.218 |
| 33 | hsa-miR-186-5p | 0.1 | 0.00102 |
| 34 | hsa-miR-188-5p | 0.14 | 0.00000363 |
| 35 | hsa-miR-195-5p | 0.142 | 0.00000279 |
| 36 | hsa-miR-320a | 0.104 | 0.000604 |
| 37 | hsa-miR-296-5p | 0.059 | 0.0511 |
| 38 | hsa-miR-130b-3p | 0.061 | 0.0461 |
| 39 | hsa-miR-376c-3p | 0.083 | 0.00608 |
| 40 | hsa-miR-377-3p | 0.112 | 0.000231 |
| 41 | hsa-miR-381-3p | 0.237 | 2.57E-15 |
| 42 | hsa-miR-323a-3p | 0.037 | 0.224 |
| 43 | hsa-miR-409-5p | 0.015 | 0.627 |
| 44 | hsa-miR-485-5p | 0.143 | 0.00000224 |
| 45 | hsa-miR-486-5p | 0.097 | 0.00137 |
| 46 | hsa-miR-146b-5p | 0.263 | 1.14E-18 |
| 47 | hsa-miR-493-5p | 0.013 | 0.669 |
| 48 | hsa-miR-494-3p | 0.063 | 0.0376 |
| 49 | hsa-miR-496 | 0.085 | 0.00487 |
| 50 | hsa-miR-497-5p | 0.101 | 0.000837 |
| 51 | hsa-miR-181d-5p | 0.166 | 3.49E-08 |
| 52 | hsa-miR-498 | 0.08 | 0.00837 |
| 53 | hsa-miR-524-5p | 0.044 | 0.152 |
| 54 | hsa-miR-520d-5p | 0.096 | 0.00154 |
| 55 | hsa-miR-503-5p | 0.043 | 0.161 |
| 56 | hsa-miR-505-3p | 0.173 | 8.82E-09 |
| 57 | hsa-miR-506-3p | 0.001 | 0.969 |
| 58 | hsa-miR-510-5p | 0.075 | 0.014 |
| 59 | hsa-miR-545-3p | 0.014 | 0.644 |
| 60 | hsa-miR-574-3p | 0.001 | 0.967 |
| 61 | hsa-miR-579-3p | 0.094 | 0.00196 |
| 62 | hsa-miR-582-5p | 0.114 | 0.000159 |
| 63 | hsa-miR-411-5p | 0.124 | 0.0000413 |
| 64 | hsa-miR-654-5p | 0.015 | 0.611 |
| 65 | hsa-miR-655-3p | 0.11 | 0.000297 |
| 66 | hsa-miR-199b-3p | 0.015 | 0.621 |
| 67 | hsa-miR-214-5p | 0.101 | 0.000862 |
| 68 | hsa-miR-362-3p | 0.166 | 3.62E-08 |
| 69 | hsa-miR-151a-5p | 0.027 | 0.369 |
| 70 | hsa-miR-488-3p | 0.244 | 3.6E-16 |
| 71 | hsa-miR-532-3p | 0.068 | 0.0261 |
| 72 | hsa-miR-455-3p | 0.095 | 0.00169 |
| 73 | hsa-miR-541-3p | 0.081 | 0.00786 |
| 74 | hsa-miR-665 | 0.044 | 0.148 |
| 75 | hsa-miR-873-5p | 0.084 | 0.00572 |
| 76 | hsa-miR-301b-3p | 0.042 | 0.171 |
| 77 | hsa-miR-934 | 0.391 | 4.99E-41 |
| 78 | hsa-miR-320b | 0.047 | 0.124 |
| 79 | hsa-miR-320c | 0.139 | 0.000004 |
| 80 | hsa-miR-1287-5p | 0.097 | 0.00142 |
| 81 | hsa-miR-1294 | 0.034 | 0.259 |
| 82 | hsa-miR-1247-5p | 0.116 | 0.000122 |
| 83 | hsa-miR-1276 | 0.119 | 0.0000798 |
| 84 | hsa-miR-1197 | 0.061 | 0.0443 |
| 85 | hsa-miR-320d | 0.083 | 0.00614 |
| 86 | hsa-miR-1913 | 0.061 | 0.0436 |
| 87 | hsa-miR-2116-3p | 0.16 | 0.000000119 |
| 88 | hsa-miR-2355-5p | 0.123 | 0.0000481 |
| 89 | hsa-miR-3194-3p | 0.035 | 0.254 |
| 90 | hsa-miR-1343-3p | 0.029 | 0.347 |
| 91 | hsa-miR-5010-5p | 0.058 | 0.0552 |
| 92 | hsa-miR-5194 | 0.032 | 0.291 |
| 93 | hsa-miR-16-5p | -0.117 | 0.000113 |
| 94 | hsa-miR-7-5p | -0.112 | 0.000206 |
| 95 | hsa-miR-200b-3p | -0.015 | 0.615 |
| 96 | hsa-miR-23b-3p | -0.017 | 0.581 |
| 97 | hsa-miR-124-3p | -0.04 | 0.19 |
| 98 | hsa-miR-125a-5p | -0.096 | 0.00156 |
| 99 | hsa-miR-149-5p | -0.282 | 2.48E-21 |
| 100 | hsa-miR-200c-3p | -0.057 | 0.0601 |
| 101 | hsa-miR-301a-3p | -0.056 | 0.0659 |
| 102 | hsa-miR-342-3p | -0.223 | 1.17E-13 |
| 102 | hsa-miR-148b-3p | -0.194 | 1.06E-10 |
| 104 | hsa-miR-331-3p | -0.207 | 6.29E-12 |
| 105 | hsa-miR-324-5p | -0.022 | 0.472 |
| 106 | hsa-miR-324-3p | -0.019 | 0.536 |
| 107 | hsa-miR-338-3p | -0.119 | 0.0000847 |
| 108 | hsa-miR-339-5p | -0.094 | 0.00193 |
| 109 | hsa-miR-335-5p | -0.041 | 0.179 |
| 110 | hsa-miR-424-5p | 0.072 | 0.0178 |
| 111 | hsa-miR-429 | -0.045 | 0.134 |
| 112 | hsa-miR-329-3p | 0.088 | 0.00383 |
| 113 | hsa-miR-483-3p | -0.002 | 0.942 |
| 114 | hsa-miR-489-3p | -0.039 | 0.196 |
| 115 | hsa-miR-490-3p | -0.011 | 0.714 |
| 116 | hsa-miR-491-5p | -0.041 | 0.179 |
| 117 | hsa-miR-615-3p | -0.06 | 0.0481 |
| 118 | hsa-miR-625-5p | -0.2 | 3.04E-11 |
| 119 | hsa-miR-642a-5p | -0.183 | 1.37E-09 |
| 120 | hsa-miR-454-3p | -0.065 | 0.0315 |
| 121 | hsa-miR-193a-5p | -0.12 | 0.0000768 |
| 122 | hsa-miR-423-5p | -0.047 | 0.122 |
| 123 | hsa-miR-589-5p | -0.097 | 0.00139 |
| 124 | hsa-miR-616-3p | -0.044 | 0.145 |
| 125 | hsa-miR-624-3p | -0.013 | 0.665 |
| 126 | hsa-miR-708-5p | -0.011 | 0.728 |
| 127 | hsa-miR-760 | -0.101 | 0.000825 |
| 128 | hsa-miR-1224-5p | -0.075 | 0.0135 |
| 129 | hsa-miR-449c-5p | -0.102 | 0.000766 |
| 130 | hsa-miR-3150b-3p | -0.076 | 0.0118 |
| 131 | hsa-miR-374c-5p | -0.08 | 0.00834 |
| 132 | hsa-miR-4739 | -0.063 | 0.0368 |
| 133 | hsa-miR-4756-5p | -0.085 | 0.00506 |
| 134 | hsa-miR-5590-3p | -0.042 | 0.17 |
| 135 | hsa-miR-766-5p | -0.001 | 0.972 |
| 136 | hsa-miR-1277-5p | -0.015 | 0.629 |
| 137 | hsa-miR-328-3p | -0.12 | 0.0000718 |

**Table S2. Correlation of miRNA-MCM2 pairs identified by ENCORI database.**

| **No.** | **miRNA** | **Coefficient-R** | **P value** |
| --- | --- | --- | --- |
| 1 | hsa-miR-15a-5p | 0.156 | 2.52E-07 |
| 2 | hsa-miR-16-5p | 0.31 | 1.23E-25 |
| 3 | hsa-miR-31-5p | 0.063 | 3.84E-02 |
| 4 | hsa-miR-103a-3p | 0.111 | 2.37E-04 |
| 5 | hsa-miR-107 | 0.227 | 3.42E-14 |
| 6 | hsa-miR-197-3p | 0.355 | 1.75E-33 |
| 7 | hsa-miR-183-5p | 0.199 | 3.63E-11 |
| 8 | hsa-miR-124-3p | 0.017 | 5.84E-01 |
| 9 | hsa-miR-138-5p | 0.137 | 5.70E-06 |
| 10 | hsa-miR-193a-3p | 0.076 | 1.22E-02 |
| 11 | hsa-miR-330-3p | 0.008 | 7.95E-01 |
| 12 | hsa-miR-326 | 0.016 | 5.95E-01 |
| 13 | hsa-miR-324-5p | 0.312 | 7.61E-26 |
| 14 | hsa-miR-338-3p | 0.039 | 1.95E-01 |
| 15 | hsa-miR-424-5p | 0.118 | 9.79E-05 |
| 16 | hsa-miR-193b-3p | 0.093 | 2.21E-03 |
| 17 | hsa-miR-503-5p | 0.252 | 3.23E-17 |
| 18 | hsa-miR-506-3p | 0.081 | 7.73E-03 |
| 19 | hsa-miR-545-3p | 0.076 | 1.25E-02 |
| 20 | hsa-miR-92b-3p | 0.074 | 1.53E-02 |
| 21 | hsa-miR-641 | 0.072 | 1.79E-02 |
| 22 | hsa-miR-362-3p | 0.072 | 1.77E-02 |
| 23 | hsa-miR-330-5p | 0.109 | 3.03E-04 |
| 24 | hsa-miR-501-3p | 0.357 | 5.92E-34 |
| 25 | hsa-miR-502-3p | 0.234 | 5.36E-15 |
| 26 | hsa-miR-628-5p | 0.145 | 1.57E-06 |
| 27 | hsa-miR-873-5p | 0.074 | 1.49E-02 |
| 28 | hsa-miR-1301-3p | 0.406 | 2.91E-44 |
| 29 | hsa-miR-1286 | 0.111 | 2.42E-04 |
| 30 | hsa-miR-1270 | 0.077 | 1.17E-02 |
| 31 | hsa-miR-3187-3p | 0.208 | 4.13E-12 |
| 32 | hsa-miR-3194-5p | 0.131 | 1.47E-05 |
| 33 | hsa-miR-2355-3p | 0.183 | 1.17E-09 |
| 34 | hsa-miR-3613-5p | 0.199 | 3.74E-11 |
| 35 | hsa-miR-676-3p | 0.013 | 6.80E-01 |
| 36 | hsa-miR-3194-3p | 0.043 | 1.60E-01 |
| 37 | hsa-miR-1343-3p | 0.156 | 2.47E-07 |
| 38 | hsa-miR-4701-5p | 0.036 | 2.36E-01 |
| 39 | hsa-miR-5000-3p | 0.138 | 5.29E-06 |
| 40 | hsa-miR-1306-5p | 0.294 | 5.13E-23 |
| 41 | hsa-miR-139-5p | -0.21 | 2.73E-12 |
| 42 | hsa-miR-34a-5p | -0.02 | 5.08E-01 |
| 43 | hsa-miR-214-3p | -0.155 | 2.98E-07 |
| 44 | hsa-miR-195-5p | -0.228 | 2.62E-14 |
| 45 | hsa-miR-296-5p | -0.047 | 1.25E-01 |
| 46 | hsa-miR-370-3p | -0.083 | 6.44E-03 |
| 47 | hsa-miR-380-3p | -0.115 | 1.44E-04 |
| 48 | hsa-miR-335-5p | -0.085 | 4.97E-03 |
| 49 | hsa-miR-449a | -0.131 | 1.57E-05 |
| 50 | hsa-miR-329-3p | -0.153 | 3.93E-07 |
| 51 | hsa-miR-489-3p | -0.135 | 8.66E-06 |
| 52 | hsa-miR-491-5p | -0.03 | 3.24E-01 |
| 53 | hsa-miR-494-3p | -0.151 | 5.77E-07 |
| 54 | hsa-miR-497-5p | -0.111 | 2.40E-04 |
| 55 | hsa-miR-510-5p | -0.06 | 4.84E-02 |
| 56 | hsa-miR-653-5p | -0.123 | 4.57E-05 |
| 57 | hsa-miR-140-3p | -0.036 | 2.31E-01 |
| 58 | hsa-miR-2681-3p | -0.025 | 4.11E-01 |
| 59 | hsa-miR-3690 | -0.048 | 1.11E-01 |
| 60 | hsa-miR-4731-5p | -0.002 | 9.41E-01 |
| 61 | hsa-miR-5047 | -0.024 | 4.21E-01 |
| 62 | hsa-miR-212-5p | -0.016 | 5.94E-01 |

**Table S3. Correlation of miRNA-MCM4 pairs identified by ENCORI database.**

| **No.** | **miRNA** | **Coefficient-R** | **P value** |
| --- | --- | --- | --- |
| 1 | hsa-miR-17-5p | 0.486 | 1.52E-65 |
| 2 | hsa-miR-20a-5p | 0.343 | 2.36E-31 |
| 3 | hsa-miR-23a-3p | 0.251 | 5.18E-17 |
| 4 | hsa-miR-27a-3p | 0.108 | 3.87E-04 |
| 5 | hsa-miR-93-5p | 0.408 | 7.88E-45 |
| 6 | hsa-miR-103a-3p | 0.048 | 1.16E-01 |
| 7 | hsa-miR-106a-5p | 0.325 | 3.96E-28 |
| 8 | hsa-miR-107 | 0.23 | 1.88E-14 |
| 9 | hsa-miR-192-5p | 0.21 | 2.80E-12 |
| 10 | hsa-miR-129-5p | 0.078 | 1.01E-02 |
| 11 | hsa-miR-215-5p | 0.041 | 1.78E-01 |
| 12 | hsa-miR-23b-3p | 0.212 | 1.61E-12 |
| 13 | hsa-miR-27b-3p | 0.077 | 1.13E-02 |
| 14 | hsa-miR-128-3p | 0.216 | 5.66E-13 |
| 15 | hsa-miR-140-5p | 0.141 | 3.20E-06 |
| 16 | hsa-miR-106b-5p | 0.389 | 1.28E-40 |
| 17 | hsa-miR-135b-5p | 0.265 | 7.54E-19 |
| 18 | hsa-miR-338-3p | 0.011 | 7.11E-01 |
| 19 | hsa-miR-20b-5p | 0.159 | 1.42E-07 |
| 20 | hsa-miR-512-3p | 0.129 | 2.16E-05 |
| 21 | hsa-miR-526b-3p | 0.083 | 6.11E-03 |
| 22 | hsa-miR-519b-3p | 0.046 | 1.29E-01 |
| 23 | hsa-miR-520b | 0.113 | 1.81E-04 |
| 24 | hsa-miR-520c-3p | 0.109 | 3.42E-04 |
| 25 | hsa-miR-524-5p | 0.009 | 7.58E-01 |
| 26 | hsa-miR-520d-5p | 0.018 | 5.56E-01 |
| 27 | hsa-miR-519a-3p | 0.221 | 1.59E-13 |
| 28 | hsa-miR-545-3p | 0.061 | 4.49E-02 |
| 29 | hsa-miR-552-3p | 0.074 | 1.46E-02 |
| 30 | hsa-miR-584-5p | 0.262 | 1.69E-18 |
| 31 | hsa-miR-588 | 0.075 | 1.30E-02 |
| 32 | hsa-miR-652-3p | 0.191 | 2.39E-10 |
| 33 | hsa-miR-660-5p | 0.143 | 2.38E-06 |
| 34 | hsa-miR-130a-5p | 0.081 | 7.43E-03 |
| 35 | hsa-miR-340-5p | 0.096 | 1.58E-03 |
| 36 | hsa-miR-532-3p | 0.335 | 8.61E-30 |
| 37 | hsa-miR-875-5p | 0.01 | 7.38E-01 |
| 38 | hsa-miR-216b-5p | 0.063 | 3.80E-02 |
| 39 | hsa-miR-1271-5p | 0.144 | 1.89E-06 |
| 40 | hsa-miR-1301-3p | 0.393 | 2.35E-41 |
| 41 | hsa-miR-1286 | 0.083 | 6.34E-03 |
| 42 | hsa-miR-1251-5p | 0.055 | 7.18E-02 |
| 43 | hsa-miR-2682-5p | 0.054 | 7.28E-02 |
| 44 | hsa-miR-3163 | 0.011 | 7.25E-01 |
| 45 | hsa-miR-514b-5p | 0.019 | 5.39E-01 |
| 46 | hsa-miR-4731-5p | 0.023 | 4.42E-01 |
| 47 | hsa-miR-5000-3p | 0.131 | 1.59E-05 |
| 48 | hsa-miR-216a-5p | -0.063 | 3.70E-02 |
| 49 | hsa-miR-135a-5p | -0.092 | 2.30E-03 |
| 50 | hsa-miR-143-3p | -0.072 | 1.73E-02 |
| 51 | hsa-miR-299-3p | -0.139 | 4.64E-06 |
| 52 | hsa-miR-373-3p | -0.028 | 3.55E-01 |
| 53 | hsa-miR-346 | -0.045 | 1.39E-01 |
| 54 | hsa-miR-515-5p | -0.017 | 5.82E-01 |
| 55 | hsa-miR-519e-5p | -0.009 | 7.67E-01 |
| 56 | hsa-miR-654-5p | -0.16 | 1.28E-07 |
| 57 | hsa-miR-214-5p | -0.147 | 1.25E-06 |
| 58 | hsa-miR-140-3p | -0.074 | 1.48E-02 |
| 59 | hsa-miR-541-3p | -0.168 | 2.45E-08 |
| 60 | hsa-miR-513c-5p | -0.022 | 4.69E-01 |
| 61 | hsa-miR-1197 | -0.013 | 6.76E-01 |
| 62 | hsa-miR-23c | -0.032 | 2.99E-01 |

**Table S4. Correlation of miRNA-MCM5 pairs identified by ENCORI database.**

| **No.** | **miRNA** | **Coefficient-R** | **P value** |
| --- | --- | --- | --- |
| 1 | hsa-miR-28-5p | 0.294 | 4.34E-23 |
| 2 | hsa-miR-103a-3p | 0.101 | 0.000908 |
| 3 | hsa-miR-107 | 0.216 | 6.55E-13 |
| 4 | hsa-miR-187-3p | 0.161 | 9.07E-08 |
| 5 | hsa-miR-217 | 0.074 | 0.0149 |
| 6 | hsa-miR-141-3p | 0.159 | 0.000000131 |
| 7 | hsa-miR-143-3p | 0.015 | 0.621 |
| 8 | hsa-miR-200a-3p | 0.089 | 0.00342 |
| 9 | hsa-miR-338-3p | 0.028 | 0.357 |
| 10 | hsa-miR-376b-3p | 0.036 | 0.23 |
| 11 | hsa-miR-503-5p | 0.157 | 0.000000215 |
| 12 | hsa-miR-545-3p | 0.074 | 0.0144 |
| 13 | hsa-miR-132-5p | 0.006 | 0.831 |
| 14 | hsa-miR-362-3p | 0.204 | 1.26E-11 |
| 15 | hsa-miR-330-5p | 0.066 | 0.0291 |
| 16 | hsa-miR-423-5p | 0.111 | 0.000234 |
| 17 | hsa-miR-708-5p | 0.05 | 0.102 |
| 18 | hsa-miR-877-5p | 0.237 | 2.61E-15 |
| 19 | hsa-miR-543 | 0.003 | 0.911 |
| 20 | hsa-miR-1286 | 0.106 | 0.000463 |
| 21 | hsa-miR-1287-5p | 0.129 | 0.0000193 |
| 22 | hsa-miR-1197 | 0.017 | 0.585 |
| 23 | hsa-miR-3139 | 0.061 | 0.0435 |
| 24 | hsa-miR-3200-3p | 0.268 | 2.59E-19 |
| 25 | hsa-miR-212-5p | 0.048 | 0.111 |
| 26 | hsa-miR-4770 | -0.089 | 0.00346 |
| 27 | hsa-miR-139-5p | -0.081 | 7.77E-03 |
| 28 | hsa-miR-10a-5p | -0.257 | 7.67E-18 |
| 29 | hsa-miR-10b-5p | -0.305 | 9.49E-25 |
| 30 | hsa-miR-183-5p | -0.028 | 3.65E-01 |
| 31 | hsa-miR-214-3p | -0.107 | 4.00E-04 |
| 32 | hsa-miR-191-5p | -0.069 | 2.22E-02 |
| 33 | hsa-miR-184 | -0.153 | 4.18E-07 |
| 34 | hsa-miR-376a-3p | -0.065 | 3.10E-02 |
| 35 | hsa-miR-326 | -0.079 | 9.16E-03 |
| 36 | hsa-miR-329-3p | -0.112 | 2.24E-04 |
| 37 | hsa-miR-432-5p | -0.116 | 1.29E-04 |
| 38 | hsa-miR-654-5p | -0.147 | 1.18E-06 |
| 39 | hsa-miR-628-5p | -0.006 | 8.33E-01 |
| 40 | hsa-miR-541-3p | -0.111 | 2.47E-04 |
| 41 | hsa-miR-3129-5p | -0.042 | 1.69E-01 |

**Table S5. Correlation of miRNA-MCM6 pairs identified by ENCORI database.**

| **No.** | **miRNA** | **Coefficient-R** | **P value** |
| --- | --- | --- | --- |
| 1 | hsa-miR-199a-3p | 0.032 | 2.96E-01 |
| 2 | hsa-miR-223-3p | 0.097 | 1.31E-03 |
| 3 | hsa-miR-141-3p | 0.171 | 1.34E-08 |
| 4 | hsa-miR-200a-3p | 0.036 | 2.36E-01 |
| 5 | hsa-miR-373-3p | 0.011 | 7.12E-01 |
| 6 | hsa-miR-323a-3p | 0.093 | 2.25E-03 |
| 7 | hsa-miR-326 | 0.102 | 7.38E-04 |
| 8 | hsa-miR-512-3p | 0.175 | 6.01E-09 |
| 9 | hsa-miR-520e | 0.183 | 1.23E-09 |
| 10 | hsa-miR-520f-3p | 0.177 | 4.73E-09 |
| 11 | hsa-miR-519c-3p | 0.109 | 3.13E-04 |
| 12 | hsa-miR-520a-5p | 0.093 | 2.23E-03 |
| 13 | hsa-miR-520a-3p | 0.005 | 8.75E-01 |
| 14 | hsa-miR-519b-3p | 0.108 | 3.62E-04 |
| 15 | hsa-miR-525-5p | 0.047 | 1.21E-01 |
| 16 | hsa-miR-520b | 0.131 | 1.43E-05 |
| 17 | hsa-miR-520c-3p | 0.128 | 2.29E-05 |
| 18 | hsa-miR-520d-3p | 0.062 | 4.10E-02 |
| 19 | hsa-miR-519a-3p | 0.217 | 4.40E-13 |
| 20 | hsa-miR-671-5p | 0.212 | 1.79E-12 |
| 21 | hsa-miR-199b-3p | 0.033 | 2.78E-01 |
| 22 | hsa-miR-362-3p | 0.067 | 2.66E-02 |
| 23 | hsa-miR-330-5p | 0.156 | 2.38E-07 |
| 24 | hsa-miR-628-5p | 0.239 | 1.51E-15 |
| 25 | hsa-miR-873-5p | 0.103 | 6.43E-04 |
| 26 | hsa-miR-1343-3p | 0.183 | 1.27E-09 |
| 27 | hsa-miR-3064-5p | 0.031 | 3.03E-01 |
| 28 | hsa-miR-34a-5p | -0.04 | 1.86E-01 |
| 29 | hsa-miR-302a-3p | -0.031 | 3.00E-01 |
| 30 | hsa-miR-34c-5p | -0.175 | 6.74E-09 |
| 31 | hsa-miR-302b-3p | -0.013 | 6.64E-01 |
| 32 | hsa-miR-302d-3p | -0.021 | 4.99E-01 |
| 33 | hsa-miR-376c-3p | -0.066 | 2.98E-02 |
| 34 | hsa-miR-449a | -0.179 | 2.69E-09 |
| 35 | hsa-miR-329-3p | -0.12 | 7.16E-05 |
| 36 | hsa-miR-490-3p | -0.04 | 1.83E-01 |
| 37 | hsa-miR-494-3p | -0.117 | 1.12E-04 |
| 38 | hsa-miR-495-3p | -0.056 | 6.60E-02 |
| 39 | hsa-miR-449b-5p | -0.081 | 7.62E-03 |
| 40 | hsa-miR-542-3p | -0.087 | 3.93E-03 |
| 41 | hsa-miR-888-5p | -0.028 | 3.51E-01 |
| 42 | hsa-miR-875-5p | -0.001 | 9.86E-01 |
| 43 | hsa-miR-1197 | -0.01 | 7.40E-01 |
| 44 | hsa-miR-3129-5p | -0.031 | 3.00E-01 |
| 45 | hsa-miR-3690 | -0.009 | 7.75E-01 |
| 46 | hsa-miR-642a-3p | -0.054 | 7.37E-02 |
| 47 | hsa-miR-6512-3p | -0.008 | 7.91E-01 |

**Table S6. Correlation of miRNA-MCM7 pairs identified by ENCORI database.**

| **No.** | **miRNA** | **Coefficient-R** | **P value** |
| --- | --- | --- | --- |
| 1 | hsa-miR-103a-3p | 0.131 | 0.0000158 |
| 2 | hsa-miR-107 | 0.217 | 5.27E-13 |
| 3 | hsa-miR-124-3p | 0.004 | 0.907 |
| 4 | hsa-miR-138-5p | 0.247 | 1.49E-16 |
| 5 | hsa-miR-141-3p | 0.228 | 2.98E-14 |
| 6 | hsa-miR-200a-3p | 0.138 | 0.00000473 |
| 7 | hsa-miR-301a-3p | 0.308 | 2.94E-25 |
| 8 | hsa-miR-130b-3p | 0.384 | 1.8E-39 |
| 9 | hsa-miR-302b-3p | 0.023 | 0.448 |
| 10 | hsa-miR-302d-3p | 0.013 | 0.658 |
| 11 | hsa-miR-135b-5p | 0.33 | 5.62E-29 |
| 12 | hsa-miR-324-3p | 0.226 | 5.3E-14 |
| 13 | hsa-miR-338-3p | 0.026 | 0.392 |
| 14 | hsa-miR-520e | 0.129 | 0.0000192 |
| 15 | hsa-miR-515-5p | 0.063 | 0.0373 |
| 16 | hsa-miR-519e-5p | 0.011 | 0.721 |
| 17 | hsa-miR-519c-3p | 0.093 | 0.00216 |
| 18 | hsa-miR-520a-3p | 0.095 | 0.00167 |
| 19 | hsa-miR-519b-3p | 0.12 | 0.0000707 |
| 20 | hsa-miR-520b | 0.138 | 0.0000049 |
| 21 | hsa-miR-520c-3p | 0.131 | 0.0000142 |
| 22 | hsa-miR-524-5p | 0.033 | 0.277 |
| 23 | hsa-miR-520d-5p | 0.035 | 0.255 |
| 24 | hsa-miR-520d-3p | 0.09 | 0.00311 |
| 25 | hsa-miR-519a-3p | 0.245 | 3.04E-16 |
| 26 | hsa-miR-506-3p | 0.026 | 0.387 |
| 27 | hsa-miR-454-3p | 0.274 | 3.71E-20 |
| 28 | hsa-miR-423-5p | 0.101 | 0.000912 |
| 29 | hsa-miR-301b-3p | 0.324 | 6.06E-28 |
| 30 | hsa-miR-1301-3p | 0.295 | 3.75E-23 |
| 31 | hsa-miR-1913 | 0.069 | 0.0227 |
| 32 | hsa-miR-214-3p | -0.125 | 3.48E-05 |
| 33 | hsa-miR-135a-5p | -0.086 | 4.71E-03 |
| 34 | hsa-miR-302a-3p | -0.041 | 1.80E-01 |
| 35 | hsa-miR-302c-3p | -0.014 | 6.46E-01 |
| 36 | hsa-miR-372-3p | -0.041 | 1.78E-01 |
| 37 | hsa-miR-373-3p | -0.003 | 9.21E-01 |
| 38 | hsa-miR-335-5p | -0.055 | 7.22E-02 |
| 39 | hsa-miR-489-3p | -0.114 | 1.76E-04 |
| 40 | hsa-miR-371a-5p | -0.012 | 6.90E-01 |

**Table S7. Correlation of miRNA-MCM9 pairs identified by ENCORI database.**

| **No.** | **miRNA** | **Coefficient-R** | **P value** |
| --- | --- | --- | --- |
| 1 | hsa-miR-186-5p | 0.025 | 4.04E-01 |
| 2 | hsa-miR-375 | 0.043 | 1.53E-01 |
| 3 | hsa-miR-378g | 0.012 | 7.04E-01 |
| 4 | hsa-miR-27a-3p | -0.114 | 1.70E-04 |
| 5 | hsa-miR-27b-3p | -0.071 | 1.93E-02 |
| 6 | hsa-miR-144-3p | -0.049 | 1.10E-01 |
| 7 | hsa-miR-381-3p | -0.033 | 2.72E-01 |
| 8 | hsa-miR-490-3p | -0.009 | 7.58E-01 |
| 9 | hsa-miR-493-3p | -0.011 | 7.25E-01 |

**Table S8. Correlation of miRNA-MCM10 pairs identified by ENCORI database.**

| **No.** | **miRNA** | **Coefficient-R** | **P value** |
| --- | --- | --- | --- |
| 1 | hsa-miR-20a-5p | 0.393 | 2.66E-41 |
| 2 | hsa-miR-21-5p | 0.092 | 2.45E-03 |
| 3 | hsa-miR-27a-3p | 0.198 | 5.14E-11 |
| 4 | hsa-miR-29b-3p | 0.042 | 1.67E-01 |
| 5 | hsa-miR-192-5p | 0.283 | 2.17E-21 |
| 6 | hsa-miR-129-5p | 0.04 | 1.89E-01 |
| 7 | hsa-miR-215-5p | 0.087 | 4.11E-03 |
| 8 | hsa-miR-200b-3p | 0.064 | 3.38E-02 |
| 9 | hsa-miR-27b-3p | 0.066 | 2.98E-02 |
| 10 | hsa-miR-124-3p | 0.025 | 4.10E-01 |
| 11 | hsa-miR-142-5p | 0.298 | 1.18E-23 |
| 12 | hsa-miR-9-5p | 0.41 | 2.72E-45 |
| 13 | hsa-miR-186-5p | 0.458 | 2.09E-57 |
| 14 | hsa-miR-200c-3p | 0.203 | 1.63E-11 |
| 15 | hsa-miR-302b-3p | 0.014 | 6.39E-01 |
| 16 | hsa-miR-302c-3p | 0.03 | 3.26E-01 |
| 17 | hsa-miR-302d-3p | 0.014 | 6.53E-01 |
| 18 | hsa-miR-373-3p | 0.015 | 6.11E-01 |
| 19 | hsa-miR-330-3p | 0.074 | 1.51E-02 |
| 20 | hsa-miR-345-5p | 0.463 | 1.15E-58 |
| 21 | hsa-miR-20b-5p | 0.192 | 1.77E-10 |
| 22 | hsa-miR-429 | 0.181 | 1.89E-09 |
| 23 | hsa-miR-409-3p | 0.01 | 7.54E-01 |
| 24 | hsa-miR-512-3p | 0.195 | 8.39E-11 |
| 25 | hsa-miR-520e | 0.191 | 2.25E-10 |
| 26 | hsa-miR-519c-3p | 0.151 | 5.80E-07 |
| 27 | hsa-miR-520a-5p | 0.105 | 5.08E-04 |
| 28 | hsa-miR-525-5p | 0.069 | 2.28E-02 |
| 29 | hsa-miR-520d-3p | 0.073 | 1.60E-02 |
| 30 | hsa-miR-519a-3p | 0.272 | 8.44E-20 |
| 31 | hsa-miR-513a-5p | 0.033 | 2.77E-01 |
| 32 | hsa-miR-506-3p | 0.03 | 3.23E-01 |
| 33 | hsa-miR-556-5p | 0.03 | 3.30E-01 |
| 34 | hsa-miR-577 | 0.385 | 1.03E-39 |
| 35 | hsa-miR-641 | 0.083 | 6.52E-03 |
| 36 | hsa-miR-340-5p | 0.13 | 1.76E-05 |
| 37 | hsa-miR-885-5p | 0.131 | 1.59E-05 |
| 38 | hsa-miR-873-5p | 0.085 | 5.19E-03 |
| 39 | hsa-miR-320b | 0.158 | 1.79E-07 |
| 40 | hsa-miR-320d | 0.007 | 8.08E-01 |
| 41 | hsa-miR-26a-5p | -0.178 | 3.53E-09 |
| 42 | hsa-miR-26b-5p | -0.033 | 2.74E-01 |
| 43 | hsa-miR-29a-3p | -0.128 | 2.45E-05 |
| 44 | hsa-miR-217 | -0.037 | 2.18E-01 |
| 45 | hsa-miR-320a | -0.03 | 3.20E-01 |
| 46 | hsa-miR-29c-3p | -0.374 | 2.40E-37 |
| 47 | hsa-miR-302a-3p | -0.005 | 8.69E-01 |
| 48 | hsa-miR-299-3p | -0.102 | 7.45E-04 |
| 49 | hsa-miR-376c-3p | -0.059 | 5.04E-02 |
| 50 | hsa-miR-372-3p | -0.094 | 1.94E-03 |
| 51 | hsa-miR-381-3p | -0.108 | 3.52E-04 |
| 52 | hsa-miR-410-3p | -0.176 | 5.02E-09 |
| 53 | hsa-miR-485-3p | -0.061 | 4.55E-02 |
| 54 | hsa-miR-490-3p | -0.006 | 8.36E-01 |
| 55 | hsa-miR-520a-3p | -0.012 | 7.04E-01 |
| 56 | hsa-miR-656-3p | -0.16 | 1.11E-07 |
| 57 | hsa-miR-758-3p | -0.122 | 5.36E-05 |
| 58 | hsa-miR-665 | -0.106 | 4.45E-04 |
| 59 | hsa-miR-543 | -0.075 | 1.29E-02 |
| 60 | hsa-miR-320c | -0.051 | 9.45E-02 |
| 61 | hsa-miR-1179 | -0.044 | 1.46E-01 |
| 62 | hsa-miR-378g | -0.071 | 1.93E-02 |
| 63 | hsa-miR-5590-3p | -0.057 | 6.22E-02 |
